# Emerging drugs and combinations to treat multiple myeloma

**DOI:** 10.18632/oncotarget.19269

**Published:** 2017-07-15

**Authors:** Alessandra Larocca, Roberto Mina, Francesca Gay, Sara Bringhen, Mario Boccadoro

**Affiliations:** ^1^ Myeloma Unit, Division of Hematology, Azienda Ospedaliero-Universitaria Città della Salute e della Scienza di Torino, Torino, Italy

**Keywords:** multiple myeloma, novel agents, monoclonal antibodies

## Abstract

In the past few years, multiple targeted therapies and immunotherapies including second generation immunomodulatory drugs (pomalidomide) and proteasome inhibitors (carfilzomib, ixazomib), monoclonal antibodies and checkpoint inhibitors were approved for the treatment of myeloma or entered advanced phases of clinical testing. These agents showed significant activity in advanced myeloma and increased the available treatment strategies.

Pomalidomide is well-tolerated and effective in patients with relapsed/refractory multiple myeloma who have exhausted any possible treatment with lenalidomide and bortezomib. Carfilzomib, a second-generation proteasome inhibitor, is active as a single agent and in combination with other anti-myeloma agents. Ixazomib is the first oral proteasome inhibitor to be evaluated in myeloma and is associated with a good safety profile and anti-myeloma activity in relapsed/refractory patients, even in those refractory to bortezomib. Monoclonal antibodies and immune checkpoint inhibitors are likely to play a major role in the treatment of myeloma over the next decade.

In phase 3 studies, triplet regimens based on these agents combined with a backbone therapy (including lenalidomide, pomalidomide or bortezomib) were more efficacious than doublet regimens in patients with relapsed/refractory multiple myeloma, with limited additional toxic effects.

This paper aims to provide an overview of the recent use of these agents for the treatment of myeloma, in particular focusing on the role of multi-agent combinations.

## INTRODUCTION

Multiple myeloma (MM) is a neoplastic disease characterized by the proliferation of abnormal bone marrow plasma cells and immunoglobulin or light chain overproduction, which can cause end-organ damage.

Before 2000, the median survival of patients with newly diagnosed MM was approximately 2.5 years. First-generation novel agents, namely bortezomib, thalidomide, and lenalidomide, and the introduction of autologous stem cell transplantation (ASCT) have substantially improved overall survival (OS), which currently ranges from 5 to 7 years [1].

Nevertheless, long-term control of the disease remains still elusive, and most patients relapse or become refractory to existing therapies. Patients with disease refractory to both immunomodulatory drugs (IMiDs) and bortezomib have a median event-free-survival (EFS) and OS of only 5 and 9 months, respectively [2].

As such, the search for newer agents with different mechanisms of action to overcome drug-resistance has led to the development of second-generation IMiDs and proteasome inhibitors (PIs), histone deacetylase inhibitors (HDACs), Akt and mTOR inhibitors. In addition, monoclonal antibodies (MoAbs) have recently enriched the treatment armamentarium against MM.

Here, we will review the clinical activity of the newer generation IMiDs and PIs, HDACs and MoAbs, giving an in-depth insight of their possible application and their role when used in combination.

## SUMMARY OF THE CLINICAL RESULTS

### Second-generation immunomodulatory drug

#### Pomalidomide

Pomalidomide is a second-generation IMiD with a structure similar to thalidomide and lenalidomide. Pomalidomide exerts its antitumor activity by anti-proliferative and pro-apoptotic effects on plasma cells, by bone marrow microenvironment modulation (anti-angiogenic and anti-inflammatory effects) and by immunomodulation (increase in T and NK cell activity, suppression of regulatory T cells) [3–7].

Previous phase I and II studies of pomalidomide (2 to 4 mg), either alone or in combination with dexamethasone, showed overall response rate (ORR) ranging from 26% to 65% and a median progression-free survival (PFS) from 3 to 13 months, depending on the number of prior therapies (median 2 to 6) and the refractoriness to bortezomib and lenalidomide [8–13].

Of note, the Intergroupe Franconphone du Myelome (IFM) tested two schedules of pomalidomide-dexamethasone in a phase II study, where pomalidomide was given either for 21 days of a 28-day cycle or continuously (28/28). No differences in terms of ORR and outcomes were reported but patients receiving pomalidomide continuously reported a higher rate of infections (19% in the 21/28 arm vs 27% in the 28/28 arm) and pneumonia (7% in the 21/28 arm 19.5% in the 28/28 arm). Consequently, the investigators recommended the 21/28 schedule of pomalidomide-dexamethasone for a better marrow recovery [14].

In the phase III MM-003 trial, pomalidomide-dexamethasone (4 mg, 21/28) was formally compared to high-dose dexamethasone (HiDex) in heavily pre-treated patients mostly refractory to both lenalidomide and bortezomib after a median of 5 prior lines of therapy (Table 1). Pomalidomide-dexamethasone induced a higher ORR than HiDex (21% vs 3%; p<0.001) and significantly prolonged median PFS (4 vs 2 months; p<0.001) and OS (not reached vs 8 months, p<0.001) [15]. Major toxicities are summarized in Table 2.

**Table 1:** Efficacy of phase-III trials for relapsed or refractory multiple myeloma

Trials	MM-003 (pomalidomide-dexamethasone vs. high-dose-dexamethasone)	ENDEAVOR (carfilzomib-dexamethasone vs. bortezomib-dexamethasone)	ASPIRE (carfilzomib-lenalidomide-dexamethasone vs. lenalidomide-dexamethasone)	TOURMALINE-MM1 (ixazomib-lenalidomide-dexamethasone vs. placebo-lenalidomide-dexamethasone)	CASTOR (daratumumab-bortezomib- dexamethasone vs. bortezomib-dexamethasone)	POLLUX (daratumumab-lenalidomide- dexamethasone vs. lenalidomide-dexamethasone)	ELOQUENT-2 (elotuzumab- lenalidomide- dexamethasone vs. lenalidomide- dexamethasone)
**Overall-response-rate (%)**	31 vs. 10	77 vs. 63	87.1 vs. 66.7	78.3 vs 71.5	83 vs 63	93% vs 76%	79 vs. 66
**Median progression-free-survival (months)**	3.8 vs. 1.9HR 0.41 (95% CI, 0.32–0.53)	18.7 vs. 9.4HR 0.53 (95% CI, 0.44–0.65)	26.3 vs. 17.6HR 0.69 (95% CI, 0.57-0.83)	20.6 vs 14.7HR 0.74 (95% CI, 0.59-0.94)	NR vs 7.2HR 0.39 (95% CI, 0.28-0.53)	NAHR 0.37 (95% CI, 0.27-0.52)	19.4 vs 14.9HR 0.70 (95% CI, 0.57-0.85)

NR, not reached; NA, not available; HR, hazard ratio; CI, confidence interval.

**Table 2:** Main toxicities of novel agent-combinations in phase III trials for relapsed or refractory multiple myeloma

Trials							
Grade 3-4 Adverse events (AE)	MM-003 (pomalidomide-dexamethasone)	ENDEAVOR (carfilzomib-dexamethasone)	ASPIRE (carfilzomib-lenalidomide-dexamethasone)	TOURMALINE-MM1 (ixazomib-lenalidomide-dexamethasone)	CASTOR (daratumumab-bortezomib- dexamethasone)	POLLUX (daratumumab-lenalidomide- dexamethasone)	ELOQUENT-2 (elotuzumab- lenalidomide- dexamethasone)
**Haematological**	Anemia 33% Neutropenia 48% Thrombocitopenia 22%	Anemia 14% Neutropenia 2% Thrombocitopenia 8%	Anemia 18% Neutropenia 30% Thrombocitopenia 17%	Anemia 9% Neutropenia 22% Thrombocitopenia 19%	Anemia 14% Neutropenia 13% Thrombocitopenia 45% Lymphocitopenia 10%	Anemia Neutropenia Thrombocitopenia	Anemia 19% Neutropenia 34% Thrombocitopenia 19% Lymphocitopenia: 77%
**Non-haematological**	Pneumonia 14% Fatigue 5%	Hypertension 9% Dyspnea 5% Cardiac failure 5%	Hypertension 4% Cardiac failure 4% Dyspnea 3%	Diarrhea 6% Rash 5%	Pneumonia 8% Fatigue 6% Diarrhea 5% Infusion related reaction 5%	Pneumonia 8% Hypertension 7% Sensory peripheral neuropathy 5% Infusion related reaction 5%	Fatigue: 8% Rash: 5%

Based on the positive results obtained with this new agent, in February 2013, the Food and Drug Administration (FDA) and subsequently the European Medicines Agency (EMA) granted accelerated approval to pomalidomide for the treatment of patients with MM who have received at least two prior therapies, including lenalidomide and bortezomib, and experienced disease progression on or within 60 days of completion of the last therapy.

### Second-generation proteasome inhibitors

#### Carfilzomib

Carfilzomib is a second-generation, epoxyketone PI that binds selectively and irreversibly to the constitutive proteasome and immunoproteasome [16, 17].

This drug was initially tested as single agent in the PX-003-A1 study, where it was given twice-weekly at the dose of 27 mg/m2. Patients with relapsed/refractory MM (RRMM) who had received a median of 5 prior lines of therapy - including bortezomib, thalidomide and lenalidomide - were enrolled in the trial. The ORR was 24% and the duration of response (DOR) and OS were 8 and 16 months, respectively [18, 19]. In 2015, based on this pivotal study, carfilzomib was granted approval in the US for the treatment of patients with RRMM who had received at least 2 prior lines of therapy including bortezomib and an IMiD. The approved dose and schedule of carfilzomib is a twice-weekly, 10-min intravenous infusion on days 1, 2, 8, 9, 15, and 16 of 28-day cycles (starting dose: 20 mg/m2 [days 1 and 2 of cycle 1]; escalated to a target dose of 27 mg/m2 thereafter).

Subsequently, carfilzomib was compared with bortezomib in the relapse setting. In the randomized phase III study ENDEAVOR, carfilzomib plus dexamethasone (Kd) demonstrated a clinically meaningful and statistically significant two-fold improvement in median PFS compared with bortezomib plus dexamethasone (Vd; 19 vs 9 months; hazard ratio [HR]: 0.53; 95% confidence interval [CI]: 0.44-0.65; P<.0001). Higher and deeper responses were observed with Kd vs Vd across all cytogenetic subgroups [20, 21]. In particular, Kd had a favorable benefit-risk profile in high-risk RRMM, and was superior to Vd, regardless of baseline cytogenetic risk status [22]. More recently, Kd has demonstrated 7.6-month longer OS than Vd (median OS 47.6 months for Kd versus 40.0 for Vd, HR=0.79; 95 % CI: 0.65 – 0.96; p=0.01), regardless of prior bortezomib therapy (HR 0.75 for no prior bortezomib; HR 0.84 for prior bortezomib) [23].

The phase I-II CHAMPION-1 study evaluated escalated doses of once-weekly carfilzomib in combination with dexamethasone in patients with RRMM. Interestingly, the maximum tolerated dose (MTD) of carfilzomib was 70 mg/m2, resulting in an ORR of 77% and a median PFS of 12.6 months [24].

Because of the impressive results, the dose and schedule of carfilzomib used in the CHAMPION-1 trial (70 mg/m2) is currently being compared with the regulatory-approved carfilzomib dose and schedule (27 mg/m2 administered twice-weekly) in the ongoing phase III ARROW study [25].

#### Ixazomib

Ixazomib (MLN9708) is the first oral PI to be introduced in the treatment of MM. It is a boronic acid that is rapidly hydrolyzed in water and converts into MLN2238. The biologically active form MLN2238 inhibits the chymotrypsin-like proteolytic site of the proteasome. *in vitro* studies have shown activity of ixazomib against MM cells, even in those resistant to bortezomib [26].

In a phase I trial, single agent ixazomib showed clinical activity in 60 patients with RRMM, with 27% ORR at the MTD (2.97 mg once-weekly) [27].

A phase II trial investigated single agent ixazomib in 33 RRMM patients at the dose of 5.5 mg in 3 or 4-week schedule. Approximately two thirds of patients required the addition of dexamethasone for either suboptimal response or progression. Results with Ixazomib plus dexamethasone were promising, with an ORR of 34% and a median EFS of 11.5 months, and no differences were found according to prior exposure to bortezomib [28].

Moreover, two doses of ixazomib (4 and 5.5 mg) given once-weekly (on days 1, 8 and 15 of a 28-day cycle) combined with dexamethasone showed to be safe and effective in RRMM patients. Ixazomib at the dose of 5 mg induced deeper responses (ORR: 38% vs 52%) but resulted in a higher rate of grade ≥3 adverse events (21% vs 54%) [29].

The promising activity of ixazomib as single agent, the oral administration, and its safety profile led to investigate its role as a maintenance agent both in the transplant (NCT02181413) and in the non-transplant (NCT02312258) settings in two ongoing phase III trials.

### Monoclonal antibodies

#### Elotuzumab

Elotuzumab is a humanized monoclonal IgG1 antibody directed against human CS1 (also known as SLAMF7), a cell surface glycoprotein highly expressed on MM cells, and at a lower level on normal plasma cells, NK cells and other T-cells [30]. CS1 mediates the adhesion of MM cells to the bone marrow stromal cells, granting their proliferation and preventing apoptosis [31]. By binding CS1, elotuzumab inhibits the stimulatory effects of the bone marrow on MM cells; furthermore, it exerts anti-MM activity via ADCC mediated by NK cells [30].

The first-in-human trial of elotuzumab as single agent was conducted in 35 RRMM patients [32]. This agent appeared to be well tolerated, and the MTD was not reached at the maximum dose tested (20 mg/kg every other week). The main adverse events were infusion-related reactions (IRR), generally mild to moderate, occurring during the first dose of elotuzumab. When the protocol was amended for premedication before the infusion of elotuzumab, no grade 3-4, nor serious IRR, were reported. Despite the appealing safety profile, single agent elotuzumab did not induce objective responses, and 26.5% of patients achieved a stable disease (SD); this evidence supported further investigation of elotuzumab in combination with other novel agents in phase II and III trials.

#### Anti-CD 38 monoclonal antibodies

CD38 is a type II transmembrane glycoprotein exerting receptor-mediated adhesion and signaling functions [33, 34]. It is expressed at relatively low levels on lymphoid and myeloid cells, as well as on other non-hematological tissues, while it is highly expressed on malignant plasma cells, thus becoming a potential therapeutic target [35].

Three anti-CD38 MoAbs were recently developed: the chimeric Isatuximab (SAR650984), and the fully humanized Daratumumab (DARA) and MOR202 (MOR) [36]. Each MoAb targets a distinct epitope on CD38, with different mechanisms of action.

#### Daratumumab

Daratumumab is a fully human IgG1 MoAb targeting a specific epitope of CD38 on the surface of MM cells [36]. It exerts its anti-myeloma effect through the activation of complement-dependent cytotoxicity (CDC), antibody-dependent cell mediated cytotoxicity (ADCC) and antibody-dependent cellular phagocytosis (ADCP); furthermore, daratumumab is able to induce direct apoptosis of myeloma cells and modulation of the enzymatic activity of CD38 [36–40].

The GEN501 study was the first-in-human trial with daratumumab. In that study, the MTD of daratumumab was not reached, with dose levels up to 24 mg/kg. The ORR was 36% in heavily pre-treated patients who received daratumumab at a dose of 16 mg/kg. Efficacy was dose-related, indeed the ORR was 10% with the 8 mg/kg dose and 35% with the higher 16 mg/kg dose [41, 42].

In the phase II SIRIUS trial, daratumumab at the dose of 16 mg/kg, was tested in 106 patients with a median of 5 prior therapies; a vast majority of patients had failed lenalidomide and bortezomib, many were refractory to pomalidomide and carfilzomib. Daratumumab induced an ORR of 29%, a median PFS of 3.7 months and a median OS of 17.5 months. Of note, the ORR was 21% in patients quadruple refractory to bortezomib, lenalidomide, pomalidomide, and carfilzomib [43].

In the pooled analysis of GEN501 and Sirius trials approximately one third of patients treated with daratumumab 16 mg/kg achieved an objective response; responses were durable (median DOR: 8 months) and independent of the number of prior therapies and renal function [44]. The median PFS and OS were 4 and 20 months, respectively. A remarkable PFS difference was observed between responding (≥PR) and non-responding patients (median, 15 vs 1 month). Interestingly, a survival advantage was reported not only among responding patients (median OS NR), but also in patients with a SD or minimal response (MR) (median OS 19 months) over non-responding patients (median OS, 4 months). Any grade IRRs occurred in 48% of patients; however, only 3% were severe IRR (≥ grade 3-4). IRRs developed during the first infusion (96%), with a low rate of reoccurrence during the subsequent infusions (7%).

Single agent daratumumab was approved in the US in November 2015 by FDA, and subsequently in Europe by EMA, for patients with MM who have received at least 3 prior lines of therapy including a PI and an IMiD or who are double-refractory to a PI and an IMiD.

#### Isatuximab (SAR650984)

Isatuximab (SAR650984) is a chimeric anti-CD38 antibody generated from immunization with murine 300-19 cells transfected to express human CD38 [45]. Isatuximab induces cell death via ADCC in all the CD38+ lines tested, ADCP and CDC in *in vitro* models. It also exerts a pro-apoptotic effect.

Isatuximab was evaluated in a dose escalation study (0.3-20 mg/kg) to determine its MTD and safety profile in patients with different RR hematological malignancies. In 18 heavily pre-treated RRMM patients (median number of prior therapies of 6), who were treated with ≥10 mg/kg isatuximab, at least a PR was documented in 33% of patients, including a complete response (CR) in 11%. IRRs mainly occurred during the first infusion and were not severe in grade (1-2). The most common treatment-emergent toxicities were fatigue (53%) and nausea (35%), and the most common drug-related grade 3-4 event was pneumonia (8%).

#### Checkpoint inhibitors

Recently, the interaction between the tumor and the immune system has become a highly relevant clinical matter. Evidence has emerged that tumor cells may impair the immune host system control through different pathways, such as the T-lymphocyte associated protein 4 (CTLA-4) and programme-death 1 (PD-1), blocking immune activity by expressing the ligands of immune checkpoint receptors. MoAbs directed against ligands and the involved receptors allow the reversal of tumor-induced down-regulation of T-cells and the enhancement of the immune response against neoplastic cells [46].

Two anti-PD1 MoAbs, nivolumab and pembrolizumab, and the anti-PDL1, durvalumab, are currently under investigation in MM patients. Nivolumab as single agent (3 mg/kg) was tested in 27 RRMM patients; no objective responses were observed, however 63% of patients achieved disease control (SD) [47]. Pembrolizumab is a humanized IgG4 MoAb, directed against PD-1, blocking the interaction between PD-1 and PD-L1/PD-L2. Pembrolizumab demonstrated effective antitumor activity and manageable safety in various cancers [48].

## SUMMARY OF DRUG-COMBINATIONS

### IMiDs and PIs combinations

New drugs currently under investigation in different combinations are summarized in Table 3. The addition of the alkylating agent cyclophosphamide to pomalidomide-dexamethasone (PD) doubled the ORR, from roughly 30% to 51-65%, and prolonged PFS from approximately 4 months to 10 months, without adding significant toxicities [49, 50].

**Table 3:** New agents currently under investigation

Class	Agent	Target	Combination tested	Approval
**Proteasome inhibitor**	Carfilzomib		KTd KRd KPd K-filanesib	Carf-Rd
	Ixazomib		Ixazomib-Rd Ixazomib-Pd	Ixa-Rd
**Immunomodulatory drug**	Pomalidomide		PVD KPd Ixazomib-Pd Dara-Pd Pembrolizumab-Pd Durvaumab-Pd Elo-Pd	Poma-dex
**Monoclonal antibody**	Elotuzumab	CS1	Elo-Rd Elo-Vd Elo-Pd	Elo-Rd
	Daratumumab	CD38	Dara-Rd Dara-Vd Dara-Pd	Dara-Rd Dara single agent
	SAR	CD38	SAR-Rd SarVCD	
**Checkpoint inhibitor**	Pembrolizumab	PD-1	Pembrolizumab-Rd Pembrolizumab-Pd	
	Durvalumab	PDL-1	Durvalumab-Rd Durvalumab-Pd	

K, carfilzomib; T, thalidomide; d, dexamethasone; R, lenalidomide; carf, carfilzomib; ixa, ixazomib; poma, pomalidomide; elo, elotuzumab; dara, daratumumab; V, bortezomib. A brief review of other innovative compounds including mTOR, MEk, BRAF and Akt inhibitors, anti IL-6 and anti KIR agents.

The pro-apoptotic effect of pomalidomide proved to be enhanced by dexamethasone and PIs [12, 51]. Two trials explored the activity of pomalidomide, bortezomib and dexamethasone (PVD) in patients who had received 1–4 lines of previous therapy. In the Phase I MM-005 study the MTD of pomalidomide was established at 4 mg on days 1–14 in a 28-day cycle [51]. In the cohort of 22 patients who received intravenous bortezomib, 71% achieved at least a PR and 38% at least a very good partial response (VGPR). Neutropenia and thrombocytopenia were the most common grades 3–4 adverse events, whereas no severe neuropathy was reported. In another phase I-II study, the MTD of once-weekly bortezomib, associated with pomalidomide (4 mg on days 1-21) and dexamethasone (40 mg weekly), was 1.3 mg/m2 [52]. Overall, 94% of patients achieved at least a PR including 56% with at least a VGPR. Neutropenia (36%) was the most common severe complication. These studies provided the basis for an ongoing, randomized phase III study comparing PVD with VD (MM-007 study).

The synergistic activity of IMiDs and PIs led physicians to test carfilzomib in combinations with both lenalidomide and pomalidomide in the relapse setting.

In a phase I/II study, carfilzomib, lenalidomide, and weekly dexamethasone (KRd) was active in patients with relapsed disease; the safety profile was consistent with the known toxicity of each agent [53, 54]. The promising results obtained in the phase I/II trial led to a randomized phase 3 study (ASPIRE) evaluating carfilzomib added to standard Rd (KRd) as compared to Rd in patients with RRMM. KRd resulted in a clinically relevant 31% decrease in the risk of disease progression or death and an increase of 8.7 months in the median PFS (26.3 months in the carfilzomib group vs. 17.6 months in the control group). The 2-year OS rate was higher in the carfilzomib group (73.3% and 65.0% respectively; HR for death 0.79; 95% CI, 0.63 to 0.99; P = 0.04). The ORRs were 87.1% and 66.7% in the carfilzomib and control groups, respectively (P<0.001). Adverse events of grade 3 or higher were reported in 83.7% and 80.7% of patients in the carfilzomib and control groups, respectively; 15.3% and 17.7% of patients discontinued treatment owing to adverse events [55].

Lenalidomide is widely adopted in the upfront setting and pomalidomide proved to be effective also in patients relapsed after and/or refractory to lenalidomide. In a phase 1 study, a new 3-drug combination consisting of carfilzomib, pomalidomide, dexamethasone, was tested. Almost all patients were dual-refractory (to both lenalidomide and bortezomib). The MTD of the regimen in this heavily pretreated patient population (median of six lines of prior therapy) was carfilzomib 27 mg/m2, pomalidomide 4 mg, dexamethasone 40 mg. Grade ≥3 non hematological toxicities were congestive heart failure, pulmonary embolisms, renal failures, and pneumonia. A high response rate of 50% was observed, the median PFS was 7.2 months, and the median OS was 20.6 months [56].

Newer early phase studies are currently evaluating carfilzomib in combination with novel compounds such as the kinesin spindle protein inhibitor Filanesib or the Bruton's tyrosine kinase inhibitor Ibrutinib, in relapsed/refractory patients [56, 57].

Preclinical studies indicated a synergistic activity of ixazomib with lenalidomide [26]. A phase I/II study of ixazomib combined with lenalidomide-dexamethasone was designed to determine the safety and efficacy of this all oral combination in newly diagnosed MM patients [58]. The recommended phase II dose of Ixazomib combined with Rd was 2.23 mg/m2 on days 1,8 and 15 of a 28-day cycle, which was converted, based on pharmacokinetic analysis, to a fixed dose of 4 mg. Ixazomib-Rd showed to be well tolerated, and the most frequent toxicities were skin adverse events (17%), neutropenia (12%), fatigue (9%) and thrombocytopenia (8%). At the recommended dose of ixazomib, this combination showed a promising efficacy (ORR was 90% with ≥VGPR rate of 62%) in both transplant-eligible and -ineligible patients. These data provided the rationale for the phase 3 TOURMALINE-MM1 study assessing ixazomib-Rd versus placebo-Rd in 772 RRMM patients with 1-3 prior lines of treatment [59]. Ixazomib was given at the dose of 4 mg on days 1,8 and 15, lenalidomide 25 mg on days 1-21 and dexamethasone on days 1,8,15 and 22 of 28-day cycles. Despite similar ORR (78% vs 72%), Ixazomib-Rd significantly improved PFS as compared to Rd (median, 21 vs 15 months; HR 0.74, p=0.012), while OS data were not mature yet. Results favoring Ixazomib-Rd were consistent across all analyzed subgroups, including age, ISS, cytogenetic abnormalities and previous exposure to PIs. Ixazomib did not add significant toxicities to Rd (any ≥ grade 3 adverse events: 68% vs 61%) and the most common toxicities were hematologic: grade 3-4 thrombocytopenia was more common in the Ixazomib arm (19% vs 9%). Any grade peripheral neuropathy was reported in 28% and 21% of patients treated with Ixazomib-Rd or placebo-Rd, respectively.

Based on those results, in November 2015, ixazomib in combination with Rd was granted approval by the FDA, and subsequently, by the EMA, for the treatment of patients with RRMM who had received at least 1 prior therapy.

The high rate of double-refractory (to both lenalidomide and bortezomib) MM patients, led investigators to combine next-generation PI and IMiDs, particularly ixazomib and pomalidomide A phase I/II, dose escalation study explored ixazomib (3-4 mg on days 1,8 and 15), pomalidomide (2-4 mg on days 1-21) and dexamethasone (20-40 mg on days 1,8,15 and 22) in a 28-day cycle in double-refractory patients [60]. In 14 evaluable patients, grade 3-4 neutropenia occurred in 29% of patients, while 12% of them experienced grade 3 infections. Low-grade peripheral neuropathy was documented in 24% of patients. The ORR was 62% in 13 evaluable patients. Therefore, the combination ixazomib-pomalidomide-dexamethasone seems to be safe and effective in this subset of patients.

### Combinations including histone deacetylase inhibitors

Histone deacetylases (HDACs) are a group of enzymes involved in the epigenetic control of various processes, from cell cycle progression to angiogenesis [61–63]. HDAC inhibitors (HDACIs) intervene in the regulation of crucial events in myeloma progression, causing cell cycle arrest, up-regulating the expression of pro-apoptotic proteins, reducing anti-oxidative stress defenses and the proteasome activity [64].

Numerous HDACIs have been developed (vorinostat, panobinostat, givinostat, romidepsin and ricolinostat) and all have been tested for the treatment of myeloma patents. However, no significant activity has been reported when used as single agent [65–68].

The combined inhibition of both proteasome and aggresome protein degradation pathways has suggested HDACIs as the ideal partner for PIs. The promising results obtained in phase I and II trials adding vorinostat to bortezomib paved the way to the phase III trial comparing Vd plus vorinostat or placebo in RRMM patients; [69, 70] despite a higher ORR (56% vs 41%), no survival advantage was reported for patients receiving vorinostat [71].

A synergistic activity between panobinostat and bortezomib-dexamethasone was reported in the phase II PANORAMA 2 trial, where the three-drug combination was able to induce an objective response among 35% of heavily pre-treated RRMM patients [72]. Based on these results, the addition of panobinostat to standard Vd was formally compared in a phase III trial (PANORAMA 1) enrolling 768 RRMM patients. Despite a similar ORR (61% vs 55%), responses were deeper with panobinostat-bortezomib-dexamethasone (PanoVd) as compared to Vd (CR, 28% vs 16%); [73] this translated into a median PFS (12 vs 8, months) and OS (34 vs 30, months) advantage for patients receivng panobinostat. Interestingly, the PFS advantage observed in the PanoVd arm was even more pronounced in patients previously exposed to both PIs and IMiDs (median, 13 vs 5, months) [74]. As expected, AEs were more frequent in the PanoVd arm, and 33% of patients had to discontinue treatment against 17% in the Vd arm. Most frequent AEs were diarrhea (68%), thrombocytopenia (67%) and fatigue (57%).

Based on these results, the FDA and EMA approved panobinostat for myeloma patients after at least 2 prior treatments including IMiDs and PIs.

### Combinations including MoAbs

#### Elotuzumab

Data from preclinical models suggest a synergistic activity elotuzumab with bortezomib and lenalidomide, probably mediated through the enhancement of elotuzumab-mediated ADCC and the stimulation of NK cell activity [31, 75].

In a phase II trial in lenalidomide-naive myeloma patients with a median of 2 previous therapies, the combination of elotuzumab-lenalidomide-dexamethasone (EloRd) was feasible and effective, with 85% least PR [76].

In another study, EloRd induced 82% PR, and 32% at least a VGPR. Prior exposure to novel agents or number of prior therapies did not appear to affect response rate [77]. No DLTs occurred and the MTD was not reached.

Based on these encouraging results, a phase III trial comparing the combination EloRd versus Rd was conducted (ELOQUENT-2) [78]. Overall, 321 patients were assigned to the elotuzumab group and 325 to the control group. After a median follow-up of 24.5 months, median PFS in the elotuzumab group was 19 versus 15 months in the control group, with a relative reduction of 30% in the risk of disease progression or death (p<0.001). Of note, EloRd combination delayed the need for subsequent myeloma therapy by a median of one year compared to Rd alone [79]. The ORR in the elotuzumab group was 79% versus 66% in the control group (P<0.001). Common grade 3 or 4 adverse events in the two groups were lymphocytopenia, neutropenia, fatigue, and pneumonia. Infusion reactions occurred in 10% of patients in the elotuzumab group and were mostly grade 1 or 2.

Elotuzumab was also evaluated in combination with bortezomib in a phase I study [80]. No DLTs occurred and the MTD was not reached. Forty-eight percent and 63% of the evaluable patients achieved at least a PR and a minimal response, respectively. Adverse events were primarily grade 1-2 IRR, observed in 71% of patients. A formal comparison between elotuzumab-bortezomib-dexamethasone (EloVd) and Vd has been recently conducted among 150 RRMM patients, and half of them had previously received bortezomib [81]. Despite a similar ORR (66% vs 63%), a trend towards a better PFS was reported in favor of patients receiving EloVd as compared to those treated with Vd (median, 10 vs 7 months; HR 0.72, p=0.09). In a preliminary survival analysis, the 2-year OS was 73% with EloVd and 66% with Vd. The addition of elotuzumab to Vd did not add significant toxicities: grade 3-4 adverse events occurred in 71% of patients who received EloVd and 60% of those treated with Vd; main toxicities were infections (21% vs 13%) and thrombocytopenia (9% vs 17%).

Preliminary data from a Spanish phase II trial showed a favorable toxicity profile and promising efficacy with Elotuzumab combined with thalidomide and low-dose dexamethasone (EloTd) in the relapse setting [82]. Forty heavily pre-treated patients (3 prior regimens) received EloTd. As per protocol, cyclophosphamide was added in 28% of patients because of lack of response or due to progression. The main toxicities reported were asthenia (any grade, 35%) and peripheral edema (any grade, 25%); EloTd was able to induce an objective response in 38% of patients, with median PFS and OS of 4 and 13 months, respectively.

#### Anti-CD38

Lenalidomide improves the efficacy of MoAbs thanks to its ability to enhance ADCC and the activity of effector cells (for example, NK cells), and to upregulate CD38 expression on MM cells [83, 84].

The combination of daratumumab with lenalidomide and dexamethasone (DaraRd) was firstly tested in a phase I/II trial enrolling RRMM patients. In the expansion phase (32 patients, not lenalidomide refractory and with a median of 2 prior lines of therapy), daratumumab was administered at the dose of 20 mg/kg. The ORR was 88% with 25% of CR (22% sCR) [85].

Based on these results, DaraRd (with daratumumab 16 mg/kg) was formally compared to standard Rd in a phase III trial (POLLUX), enrolling 569 RRMM patients after at least 1 prior regimen. [86] Patients in the experimental arm had a significantly lower risk of disease progression or death as compared to patients in the standard arm (HR 0.32; p<0.001), regardless of the number of prior therapies or previous lenalidomide exposure. Of notice, a significantly higher proportion of patients in the DaraRd group reached minimal residual disease (MRD; threshold of 1 tumor cell per 10^5 white cells) than those in the Rd group (22% vs 5%; p<0.001). The addition of daratumumab to Rd did not affect the tolerability of the combination: adverse events leading to treatment discontinuation were in fact similar in the groups (7% vs 8%).

A twin phase III, randomized study (CASTOR), evaluating the benefit of the addition of daratumumab to VD in the relapse setting, has been recently published. Results are impressive: daratumumab-VD, significantly improved median PFS, with 61% reduction in the risk of progression (P<0.001) and induced a marked increase in ORR (83% vs 63%, P<0.001) and in at least VGPR rate (59% vs 29%, P<0.001). As reported in the POLLUX trial, the addition of daratumumab significantly increased the rate of MRD negativity at all the examined thresholds (1 tumor cell per 10^4, 10^5 and 10^6 white cells; p<0.001) [87, 88].

Daratumumab has been also tested in combination with next generation novel agents such as carfilzomib and pomalidomide. An ongoing phase Ib study is evaluating the triplet daratumumab, carfilzomib and dexamethasone and will clarify the role of daratumumab in association with carfilzomib. Similarly, another study assessed the role of daratumumab plus the third-generation IMiD pomalidomide (DaraPD) in 77 RRMM with 3.5 median prior lines of treatment. Preliminary results are promising, as daratumumab did not add significant toxicities to those reported with PD, except for IRRs, which occurred in 61% of patients. In 53 patients evaluable for efficacy, ORR was 58.5%, with similar results in the double refractory (lenalidomide and bortezomib) population (ORR 57.5%) [57].

Another interesting MoAb is Isatuximab (SAR650984), which has demonstrated synergistic or additive antitumor effects in combination with lenalidomide, bortezomib, carfilzomib and melphalan in mouse xenograft tumor models. Isatuximab in combination with Rd in heavily pre-treated patients (with a median of 7 prior treatments), reported an ORR of 63% at the dose of 10 mg/kg and a reduction in paraprotein of >90% was recorded in approximately one-third of patients [89]. The vast majority of patients in this study were relapsed or refractory to lenalidomide, yet the ORR was 48% in this patient subpopulation. The median PFS was 6.2 months, yet in patients who had received only 1-2 lines of prior therapy (n=7) median PFS had not been reached at data cut-off. Notably, responses were also observed in patients refractory to bortezomib, carfilzomib or pomalidomide [89].

#### Checkpoint-inhibitors

In preclinical studies, lenalidomide has been shown to enhance the efficacy of the checkpoint blockade in contrasting tumor growth [90]. This evidence provided the scientific rationale for the combination of checkpoint inhibitors (pembrolizumab, anti-PD1, and durvalumab, anti-PD-L1) with IMiDs.

In a phase I, dose escalation trial, pembrolizumab combined with Rd (PembroRd) was tested in RRMM patients, after at least 2 prior regimens; of note, 76% patients were refractory to lenalidomide while 30% were double-refractory. Most common adverse events were thrombocytopenia (28%) and neutropenia (24%). Impressively, 76% of patients achieved at least a PR, with a median DOR of 10 months [91].

An ongoing phase II trial is exploring the safety and efficacy of pembrolizumab plus pomalidomide-dexamethasone in RRMM [92]. A preliminary analysis of 24 evaluable patients previously exposed to both IMiDs and PIs showed that the combination Pembrolizumab-PD was well tolerated with most grade 3-4 adverse events being hematological. No IRRs were reported. Autoimmune toxicities were reported: hypotiroidism in 8%, transaminitis in 8% and pneumonitis in 4% of the studied population. This combination also showed a good efficacy, with at least PR of 50%.

## INTEGRATION OF DRUG COMBINATIONS INTO CLINICAL CARE AND FUTURE DIRECTIONS

At diagnosis, ASCT significantly improved PFS as compared with bortezomib- and lenalidomide-based regimens without transplant, even if a survival benefit was not observed in all trials. ASCT-eligible patients usually receive a 3-drug induction treatment with IMID-PI and dexamethasone. Bortezomib-thalidomide-dexamethasone (VTD) and bortezomib-lenalidomide-dexamethasone (VRD) represent, to date, the standard induction approaches for transplant-eligible patients;[93–95] however, new combinations are currently under investigation as induction options in these patients.

Preliminary results showed that induction treatment with ixazomib-lenalidomide-dexamethasone (IRD)[96] was very well tolerated (no grade 3-4 neuropathy, cardiac, liver or renal toxicities) and led to a at least a VGPR rate of 38%. After induction with Carfilzomib-Thalidomide-dexamethasone (KTd)[97] or plus lenalidomide and dexamethasone (KRd)[98] more than 60% of patients achieved at least a VGPR, but cardiovascular toxicities have been reported.

Four-drug combinations may become the future strategy, with the incorporation of monoclonal antibodies as part of first-line and maintenance therapy to further improve the depth and duration of response, both in young and elderly patients.

In the next few years, myeloma patients will have several therapeutic options at relapse; treatment choice should take into account patient's fitness, disease aggressiveness, previous lines of therapies and sensibility or refractoriness to different compounds. When feasible, a multi-drug approach should be preferred.

Carfilzomib showed to be more effective than bortezomib, with a lower incidence of neuropathy and an increased cardiovascular toxicity in about 5% of patients. In combination with lenalidomide, KRd was active in patients previously exposed to bortezomib or lenalidomide and could be indicated in patients with an aggressive relapse [55]. Promising combinations with HDAC inhibitors, pomalidomide and several other compounds are being explored.

Ixazomib exerts a substantial activity both alone and in combination with IMIDs [59]. Its route of administration, (oral) and schedule (once-weekly) make ixazomib an attractive option for elderly patients. Similarly, this agent might be attractive for maintenance therapy and, at relapse, Ixazomib combined with Rd may be particularly indicated for patients with indolent relapse.

Despite being active also as single agent, anti-CD38 antibodies combined with other novel agents, such as lenalidomide and bortezomib, led to unprecedented results [86, 87]. For this reason daratumumab is likely to become part of the backbone treatment of both newly diagnosed and RRMM patients in the next future.

At relapse, the addition of elotuzumab to myeloma regimens, particularly to Rd, significantly improved PFS and time to next therapy [77]. Currently, elotuzumab could be considered for patients not treated with lenalidomide maintenance. In the future elotuzumab could be a preferable alternative as maintenance agent or in combination with current standard treatment at relapse, particularly in case of non-aggressive relapse.

Pomalidomide may be combined with MoAbs given the synergistic immunologic activity and the potency of this combination;[57] particularly when patients become refractory to lenalidomide or bortezomib.

A special consideration should be given to high-risk patients, who have benefited less from new drugs over the past decade. Based on data available today, combining a PI – such as carfilzomib or ixazomib - with lenalidomide and dexamethasone at least partially reverts the adverse effect of t(4;14) and del17p at diagnosis [99, 100]. Double ASCT plus bortezomib may improve outcome in patients with both t(4;14) and del17p. Next generation agents seem to overcome poor risk cytogenetics, but most of data was obtained in non-randomized studies and longer follow-up is needed.

Unfortunately, in elderly patients there are no data suggesting an improved outcome in high-risk patients.

In conclusion, new agents, particularly when combined together, can overcome drug resistance, improve outcome and be a valuable strategy to address the clonal heterogeneity of MM.

A variety of new drugs, with different targets and mechanisms of action, have been developed and are currently under investigation in early phase trials; some of these compounds already showed an objective anti-myeloma activity within the context of clinical trials (Table 4). It is therefore likely that multi-drug combinations will become a standard therapeutic approach for both newly diagnosed as well as relapsed/refractory myeloma.

**Table 4:** New anti-myeloma compounds in early-phase studies in RRMM patients

Class	Mechanism of action	Drug	Clinical studies
mTOR inhibitors	Regulation of cell growth, protein synthesis and cell progression [101]		
		Everolimus	• Phase 1, single agent [102] • Phase 1, in combination with lenalidomide [103]
		Temsirolimus	• Phase 1, in combination with lenalidomide [104] • Phase 1/2, in combination with bortezomib [105]
MEK1/2 inhibitor	Inhibition of cell growth [106]		
		Trametinib	• Retrospective data, 2 • Phase 1, in combination with afuresertib in solid tumors and MM [107]
BRAF inhibitor	Inhibition of the costitutionally activated NRAS–BRAF–MEK–ERK pathway that leads to excessive cellular growth survival [108]		
		Vemurafenib	• Retrospective data in combination with cometinib [109] • Phase 2, single agent in patients BRAF V600m-positive [110]
AKT inhibitor	Inhibition of cell growth, apoptosis promotion [111–113]		
		Afuresertib	• Phase 1, in combination with trametinib in solid tumors and MM [107] • Phase 1, single agent in advanced hematologic malignancies including MM [114]
anti IL-6	Promoting cell-apoptosis by blocking IL-6 through a chimeric anti-IL6 monoclonal antibody [115]		
		Siltuximab	• Phase 2, single agent or in combination with dexamethasone [116] • Phase 2, randomized, in combination with bortezomib or placebo [117] • Phase 1, in combination with bortezomib and dexamethasone [118]

## References

[1] van de Donk NW, Lokhorst HM (2013). New developments in the management and treatment of newly diagnosed and relapsed/refractory multiple myeloma patients. Expert Opin Pharmacother.

[2] Kumar SK, Lee JH, Lahuerta JJ, Morgan G, Richardson PG, Crowley J, Haessler J, Feather J, Hoering A, Moreau P, LeLeu X, Hulin C, Klein SK (2012). Risk of progression and survival in multiple myeloma relapsing after therapy with IMiDs and bortezomib: a multicenter international myeloma working group study. Leukemia.

[3] Quach H, Ritchie D, Stewart AK, Neeson P, Harrison S, Smyth MJ, Prince HM (2010). Mechanism of action of immunomodulatory drugs (IMiDS) in multiple myeloma. Leukemia.

[4] Verhelle D, Corral LG, Wong K, Mueller JH, Moutouh-de Parseval L, Jensen-Pergakes K, Schafer PH, Chen R, Glezer E, Ferguson GD, Lopez-Girona A, Muller GW, Brady HA (2007). Lenalidomide and CC-4047 inhibit the proliferation of malignant B cells while expanding normal CD34+ progenitor cells. Cancer Res.

[5] Corral LG, Haslett PA, Muller GW, Chen R, Wong LM, Ocampo CJ, Patterson RT, Stirling DI, Kaplan G (1999). Differential cytokine modulation and T cell activation by two distinct classes of thalidomide analogues that are potent inhibitors of TNF-alpha. J Immunol.

[6] Görgün G, Calabrese E, Soydan E, Hideshima T, Perrone G, Bandi M, Cirstea D, Santo L, Hu Y, Tai YT, Nahar S, Mimura N, Fabre C (2010). Immunomodulatory effects of lenalidomide and pomalidomide on interaction of tumor and bone marrow accessory cells in multiple myeloma. Blood.

[7] Anderson G, Gries M, Kurihara N, Honjo T, Anderson J, Donnenberg V, Donnenberg A, Ghobrial I, Mapara MY, Stirling D, Roodman D, Lentzsch S (2006). Thalidomide derivative CC-4047 inhibits osteoclast formation by down-regulation of PU.1. Blood.

[8] Schey SA, Fields P, Bartlett JB, Clarke IA, Ashan G, Knight RD, Streetly M, Dalgleish AG (2004). Phase I study of an immunomodulatory thalidomide analog, CC-4047, in relapsed or refractory multiple myeloma. J Clin Oncol.

[9] Streetly MJ, Gyertson K, Daniel Y, Zeldis JB, Kazmi M, Schey SA (2008). Alternate day pomalidomide retains anti-myeloma effect with reduced adverse events and evidence of in vivo immunomodulation. Br J Haematol.

[10] Richardson PG, Siegel D, Baz R, Kelley SL, Munshi NC, Laubach J, Sullivan D, Alsina M, Schlossman R, Ghobrial IM, Doss D, Loughney N, McBride L (2013). Phase 1 study of pomalidomide MTD, safety, and efficacy in patients with refractory multiple myeloma who have received lenalidomide and bortezomib. Blood.

[11] Richardson PG, Siegel DS, Vij R, Hofmeister CC, Baz R, Jagannath S, Chen C, Lonial S, Jakubowiak A, Bahlis N, Song K, Belch A, Raje N (2014). Pomalidomide alone or in combination with low-dose dexamethasone in relapsed and refractory multiple myeloma: a randomized phase 2 study. Blood.

[12] Lacy MQ, Hayman SR, Gertz MA, Dispenzieri A, Buadi F, Kumar S, Greipp PR, Lust JA, Russell SJ, Dingli D, Kyle RA, Fonseca R, Bergsagel PL (2009). Pomalidomide (CC4047) plus low-dose dexamethasone as therapy for relapsed multiple myeloma. J Clin Oncol.

[13] Lacy MQ, Kumar SK, Laplant BR, Laumann K, Gertz MA, Hayman SR, Buadi FK, Dispenzieri A, Lust JA, Russell S, Dingli D, Zeldenrust SR, Fonseca R (2012). Pomalidomide plus low-dose dexamethasone (Pom/Dex) in relapsed myeloma: long term follow up and factors predicing outcome in 345 patients. Blood.

[14] Leleu X, Attal M, Arnulf B, Moreau P, Traulle C, Marit G, Mathiot C, Petillon MO, Macro M, Roussel M, Pegourie B, Kolb B, Stoppa AM (2013). Pomalidomide plus low-dose dexamethasone is active and well tolerated in bortezomib and lenalidomide-refractory multiple myeloma: Intergroupe Francophone du Myélome 2009-02. Blood.

[15] Morgan G, Palumbo A, Dhanasiri S, Lee D, Weisel K, Facon T, Delforge M, Oriol A, Zaki M, Yu X, Sternas L, Jacques C, Akehurst R (2015). Overall survival of relapsed and refractory multiple myeloma patients after adjusting for crossover in the MM-003 trial for pomalidomide plus low-dose dexamethasone. Br J Haematol.

[16] Hanada M, Sugawara K, Kaneta K, Toda S, Nishiyama Y, Tomita K, Yamamoto H, Konishi M, Oki T (1992). Epoxomicin, a new antitumor agent of microbial origin. J Antibiot (Tokyo).

[17] Meng L, Mohan R, Kwok BH, Elofsson M, Sin N, Crews CM (1999). Epoxomicin, a potent and selective proteasome inhibitor, exhibits in vivo antiinflammatory activity. Proc Natl Acad Sci U S A.

[18] Siegel DS, Martin T, Wang M, Vij R, Jakubowiak AJ, Lonial S, Trudel S, Kukreti V, Bahlis N, Alsina M, Chanan-Khan A, Buadi F, Reu FJ (2012). A phase 2 study of single-agent carfilzomib (PX-171-003-A1) in patients with relapsed and refractory multiple myeloma. Blood.

[19] Jagannath S, Vij R, Stewart AK, Trudel S, Jakubowiak AJ, Reiman T, Somlo G, Bahlis N, Lonial S, Kunkel LA, Wong A, Orlowski RZ (2012). An open-label single-arm pilot phase II study (PX-171-003-A0) of low-dose, single-agent carfilzomib in patients with relapsed and refractory multiple myeloma. Clin Lymphoma Myeloma Leuk.

[20] Dimopoulos MA, Moreau P, Palumbo A, Joshua DE, Pour L, Hajek R, Facon T, Ludwig H, Oriol A, Goldschmidt H, Rosinol L, Straub J (2015). Carfilzomib and dexamethasone (Kd) vs bortezomib and dexamethasone (Vd) in patients (pts) with relapsed multiple myeloma (RMM): results from the phase III study ENDEAVOR. J Clin Oncol.

[21] Dimopoulos MA, Moreau P, Palumbo A, Joshua D, Pour L, Hájek R, Facon T, Ludwig H, Oriol A, Goldschmidt H, Rosiñol L, Straub J, Suvorov A (2016). Carfilzomib and dexamethasone versus bortezomib and dexamethasone for patients with relapsed or refractory multiple myeloma (ENDEAVOR): a randomised, phase 3, open-label, multicentre study. Lancet Oncol.

[22] Chng WJ, Goldschmidt H, Dimopoulos MA, Moreau P, Joshua D, Palumbo A, Facon T, Ludwig H, Pour L, Niesvizky R, Oriol A, Rosinol L, Suvorov A (2015). Efficacy and safety of carfilzomib and dexamethasone vs bortezomib and dexamethasone in patients with relapsed multiple myeloma based on cytogenetic risk status: subgroup analysis from the phase 3 study endeavor (NCT01568866). Blood.

[23] Siegel DS, Oriol A, Rajnics P, Minarik J, Hungria V, Lee JH, Song K, Obreja M, Aggarwal S, Hajek R (2017). Updated results from ASPIRE and ENDEAVOR, randomized, open-label, multicenter phase 3 studies of carfilzomib in patients (Pts) with relapsed/refractory multiple myeloma (RRMM). IMW.

[24] Berenson JR, Cartmell A, Bessudo A, Lyons RM, Harb W, Tzachanis D, Agajanian R, Boccia R, Coleman M, Moss RA, Rifkin RM, Patel P, Dixon S (2016). CHAMPION-1: a phase 1/2 study of once-weekly carfilzomib and dexamethasone for relapsed or refractory multiple myeloma. Blood.

[25] Berenson J, Cartmell A, Lyons R, Harb W, Tzachanis D, Agajanian R, Boccia RV, Coleman M, Moss RA, Rifkin RM, Schupp M, Dixon S, Ou Y (2015). Weekly carfilzomib with dexamethasone for patients with relapsed or refractory multiple myeloma: updated results from the phase 1/2 study champion-1 (NCT01677858). Blood.

[26] Chauhan D, Tian Z, Zhou B, Kuhn D, Orlowski R, Raje N, Richardson P, Anderson KC (2011). In vitro and in vivo selective antitumor activity of a novel orally bioavailable proteasome inhibitor MLN9708 against multiple myeloma cells. Clin Cancer Res.

[27] Kumar SK, Bensinger WI, Zimmerman TM, Reeder CB, Berenson JR, Berg D, Hui AM, Gupta N, Di Bacco A, Yu J, Shou Y, Niesvizky R (2014). Phase 1 study of weekly dosing with the investigational oral proteasome inhibitor ixazomib in relapsed/refractory multiple myeloma. Blood.

[28] Kumar SK, LaPlant B, Roy V, Reeder CB, Lacy MQ, Gertz MA, Laumann K, Thompson MA, Witzig TE, Buadi FK, Rivera CE, Mikhael JR, Bergsagel PL (2015). Phase 2 trial of ixazomib in patients with relapsed multiple myeloma not refractory to bortezomib. Blood Cancer J.

[29] Kumar SK, Laplant BR, Reeder CB, Roy V, Buadi F, Gertz MA, Laumann K, Bergsagel PL, Dispenzieri A, Kapoor A, Mikhael J, Stewart K, Hayman SR (2015). Randomized phase 2 trial of two different doses of ixazomib in patients with relapsed multiple myeloma not refractory to bortezomib. Blood.

[30] Hsi ED, Steinle R, Balasa B, Szmania S, Draksharapu A, Shum BP, Huseni M, Powers D, Nanisetti A, Zhang Y, Rice AG, van Abbema A, Wong M (2008). CS1, a potential new therapeutic antibody target for the treatment of multiple myeloma. Clin Cancer Res.

[31] Tai YT, Dillon M, Song W, Leiba M, Li XF, Burger P, Lee AI, Podar K, Hideshima T, Rice AG, van Abbema A, Jesaitis L, Caras I (2008). Anti-CS1 humanized monoclonal antibody HuLuc63 inhibits myeloma cell adhesion and induces antibody-dependent cellular cytotoxicity in the bone marrow milieu. Blood.

[32] Zonder JA, Mohrbacher AF, Singhal S, van Rhee F, Bensinger WI, Ding H, Fry J, Afar DEH, Singhal AK (2012). A phase 1, multicenter, open-label, dose escalation study of elotuzumab in patients with advanced multiple myeloma. Blood.

[33] Konopleva M, Estrov Z, Zhao S, Andreeff M, Mehta K (1998). Ligation of cell surface CD38 protein with agonistic monoclonal antibody induces a cell growth signal in myeloid leukemia cells. J Immunol.

[34] Deaglio S, Vaisitti T, Billington R, Bergui L, Omede' P, Genazzani AA, Malavasi F (2007). CD38/CD19: a lipid raft-dependent signaling complex in human B cells. Blood.

[35] Lin P, Owens R, Tricot G, Wilson CS (2004). Flow cytometric immunophenotypic analysis of 306 cases of multiple myeloma. Am J Clin Pathol.

[36] de Weers M, Tai YT, van der Veer MS, Bakker JM, Vink T, Jacobs DC, Oomen LA, Peipp M, Valerius T, Slootstra JW, Mutis T, Bleeker WK, Anderson KC (2011). Daratumumab, a novel therapeutic human CD38 monoclonal antibody, induces killing of multiple myeloma and other hematological tumors. J Immunol.

[37] Beum PV, Lindorfer MA, Peek EM, Stukenberg PT, de Weers M, Beurskens FJ, Parren PW, van de Winkel JG, Taylor RP (2011). Penetration of antibody-opsonized cells by the membrane attack complex of complement promotes Ca(2+) influx and induces streamers. Eur J Immunol.

[38] Overdijk MB, Verploegen S, Marijn B, van Egmond M, Groen RW, Martens AC, van Bueren JL, Bleeker WP (2012). Phagocytosis is a mechanism of action for daratumumab. Blood.

[39] Groen RW, van der Veer M, Hofhuis FM, van Kessel B, de Weers M, Parren PW, Lokhorst HM, Mutis TMA (2011). In vitro and in vivo efficacy of CD38 directed therapy with daratumumab in the treatment of multiple myeloma. Blood.

[40] Jansen JH, Boross P, Overdijk MB, van Bueren JJ, Parren PWI, Leusen JH (2012). Daratumumab, a human CD38 antibody induces apoptosis of myeloma tumor cells via Fc receptor-mediated crosslinking. Blood.

[41] Lokhorst HM, Laubach J, Nahi H, Plesner T, Gimsing P, Hansson M, Minnema M, Lassen UN, Krejcik J, Ahmadi T, Lisby S, Basse L, Brun NC, Richardson PG (2014). Dose-dependent efficacy of daratumumab (DARA) as monotherapy in patients with relapsed or refractory multiple myeloma (RR MM). J Clin Oncol.

[42] Lokhorst HM, Plesner T, Laubach JP, Nahi H, Gimsing P, Hansson M, Minnema MC, Lassen U, Krejcik J, Palumbo A, van de Donk NW, Ahmadi T, Khan I (2015). Targeting CD38 with daratumumab monotherapy in multiple myeloma. N Engl J Med.

[43] Lonial S, Weiss BM, Usmani SZ, Singhal S, Chari A, Bahlis NJ, Belch A, Krishnan A, Vescio RA, Mateos MV, Mazumder A, Orlowski RZ, Sutherland HJ (2016). Daratumumab monotherapy in patients with treatment-refractory multiple myeloma (SIRIUS): an open-label, randomised, phase 2 trial. Lancet.

[44] Usmani SZ, Weiss BM, Plesner T, Bahlis NJ, Belch A, Lonial S, Lokhorst HM, Voorhees PM, Richardson PG, Chari A, Sasser AK, Axel A, Feng H (2016). Clinical efficacy of daratumumab monotherapy in patients with heavily pretreated relapsed or refractory multiple myeloma. Blood.

[45] Deckert J, Wetzel MC, Bartle LM, Skaletskaya A, Goldmacher VS, Vallée F, Zhou-Liu Q, Ferrari P, Pouzieux S, Lahoute C, Dumontet C, Plesa A, Chiron M (2014). SAR650984, a novel humanized CD38-targeting antibody, demonstrates potent antitumor activity in models of multiple myeloma and other CD38+ hematologic malignancies. Clin Cancer Res.

[46] Armand P (2015). Immune checkpoint blockade in hematologic malignancies. Blood.

[47] Lesokhin AM, Ansell SM, Armand P, Scott EC, Halwani A, Gutierrez M, Millenson MM, Cohen AD, Schuster SJ, Lebovic D, Dhodapkar M, Avigan D, Chapuy B (2016). Nivolumab in patients with relapsed or refractory hematologic malignancy: preliminary results of a phase Ib study. J Clin Oncol.

[48] Homet Moreno B, Ribas A (2015). Anti-programmed cell death protein-1/ligand-1 therapy in different cancers. Br J Cancer.

[49] Larocca A, Montefusco V, Bringhen S, Rossi D, Crippa C, Mina R, Galli M, Marcatti M, La Verde G, Giuliani N, Magarotto V, Guglielmelli T, Rota-Scalabrini D (2013). Pomalidomide, cyclophosphamide, and prednisone for relapsed/refractory multiple myeloma: a multicenter phase 1/2 open-label study. Blood.

[50] Baz RC, Martin TG, Lin HY, Zhao X, Shain KH, Cho HJ, Wolf JL, Mahindra A, Chari A, Sullivan DM, Nardelli LA, Lau K, Alsina M (2016). Randomized multicenter phase 2 study of pomalidomide, cyclophosphamide, and dexamethasone in relapsed refractory myeloma. Blood.

[51] Richardson PG, Hofmeister CC, Siegel DS, Lonial S, Laubach JP, Efebera YA, Vesole DH, Nooka AK, Rosenblatt J, Raje N, Zaki MH, Hua Y, Li Y (2013). MM-005: a phase 1 trial of pomalidomide, bortezomib, and low-dose dexamethasone (PVD) in relapsed and/or refractory multiple myeloma (RRMM). Blood.

[52] Mikhael JR, Roy V, Richardson PG, Jakubowiak A, Laumann K, LaPlant BR, Dispenzieri A, Rajkumar SV, Fonseca R, Leung N, Buadi F, Bergsagel PL, Kyle R (2013). A phase I/II trial of pomalidomide, bortezomib and dexamethasone in patients with relpased or refractory multiple myeloma. Blood.

[53] Niesvizky R, Martin TG, Bensinger WI, Alsina M, Siegel DS, Kunkel LA, Wong AF, Lee S, Orlowski RZ, Wang M (2013). Phase Ib dose-escalation study (PX-171-006) of carfilzomib, lenalidomide, and low-dose dexamethasone in relapsed or progressive multiple myeloma. Clin Cancer Res.

[54] Wang M, Martin T, Bensinger W, Alsina M, Siegel DS, Kavalerchik E, Huang M, Orlowski RZ, Niesvizky R (2013). Phase 2 dose-expansion study (PX-171-006) of carfilzomib, lenalidomide, and low-dose dexamethasone in relapsed or progressive multiple myeloma. Blood.

[55] Stewart AK, Rajkumar SV, Dimopoulos MA, Masszi T, Špička I, Oriol A, Hájek R, Rosiñol L, Siegel DS, Mihaylov GG, Goranova-Marinova V, Rajnics P, Suvorov A (2015). Carfilzomib, lenalidomide, and dexamethasone for relapsed multiple myeloma. N Engl J Med.

[56] Shah JJ, Stadtmauer EA, Abonour R, Cohen AD, Bensinger WI, Gasparetto C, Kaufman JL, Lentzsch S, Vogl DT, Gomes CL, Pascucci N, Smith DD, Orlowski RZ (2015). Carfilzomib, pomalidomide, and dexamethasone for relapsed or refractory myeloma. Blood.

[57] Chari A, Lonial S, Suvannasankha A, Fay JW, Arnulf B, Ifthikharuddin JJ, Qin X, Masterson T, Nottage K, Schecter JM, Ahmadi T, Weiss B, Krishnan A, Lentzsch S (2015). Open-label, multicenter, phase 1b study of daratumumab in combination with pomalidomide and dexamethasone in patients with at least 2 lines of prior therapy and relapsed or relapsed and refractory multiple myeloma. Blood.

[58] Kumar SK, Berdeja JG, Niesvizky R, Lonial S, Laubach JP, Hamadani M, Stewart AK, Hari P, Roy V, Vescio R, Kaufman JL, Berg D, Liao E (2014). Safety and tolerability of ixazomib, an oral proteasome inhibitor, in combination with lenalidomide and dexamethasone in patients with previously untreated multiple myeloma: an open-label phase 1/2 study. Lancet Oncol.

[59] Moreau P, Masszi T, Grzasko N, Bahlis NJ, Hansson M, Pour L, Sandhu I, Ganly P, Baker BW, Jackson SR, Stoppa AM, Simpson DR, Gimsing P (2016). Oral ixazomib, lenalidomide, and dexamethasone for multiple myeloma. N Engl J Med.

[60] Voorhees PM, Mulkey F, Hassoun H, Paba-Prada CE, Efebera YA, Hoke E, Aquino G, Carlisle D, Suman V, Richardson PG (2015). Alliance A061202. a phase I/II study of pomalidomide, dexamethasone and ixazomib versus pomalidomide and dexamethasone for patients with multiple myeloma refractory to lenalidomide and proteasome inhibitor based therapy: phase I results. Blood.

[61] Jones PA, Baylin SB (2002). The fundamental role of epigenetic events in cancer. Nat Rev Genet.

[62] Smith EM, Boyd K, Davies FE (2010). The potential role of epigenetic therapy in multiple myeloma. Br J Haematol.

[63] Witt O, Deubzer HE, Milde T, Oehme I (2009). HDAC family: what are the cancer relevant targets?. Cancer Lett.

[64] Tandon N, Ramakrishnan V, Kumar SK (2016). Clinical use and applications of histone deacetylase inhibitors in multiple myeloma. Clin Pharmacol.

[65] Richardson P, Mitsiades C, Colson K, Reilly E, McBride L, Chiao J, Sun L, Ricker J, Rizvi S, Oerth C, Atkins B, Fearen I, Anderson K (2008). Phase I trial of oral vorinostat (suberoylanilide hydroxamic acid, SAHA) in patients with advanced multiple myeloma. Leuk Lymphoma.

[66] Wolf JL, Siegel D, Goldschmidt H, Hazell K, Bourquelot PM, Bengoudifa BR, Matous J, Vij R, de Magalhaes-Silverman M, Abonour R, Anderson KC, Lonial S (2012). Phase II trial of the pan-deacetylase inhibitor panobinostat as a single agent in advanced relapsed/refractory multiple myeloma. Leuk Lymphoma.

[67] Galli M, Salmoiraghi S, Golay J, Gozzini A, Crippa C, Pescosta N, Rambaldi A (2010). A phase II multiple dose clinical trial of histone deacetylase inhibitor ITF2357 in patients with relapsed or progressive multiple myeloma. Ann Hematol.

[68] Niesvizky R, Ely S, Mark T, Aggarwal S, Gabrilove JL, Wright JJ, Chen-Kiang S, Sparano JA (2011). Phase 2 trial of the histone deacetylase inhibitor romidepsin for the treatment of refractory multiple myeloma. Cancer.

[69] Badros A, Burger AM, Philip S, Niesvizky R, Kolla SS, Goloubeva O, Harris C, Zwiebel J, Wright JJ, Espinoza-Delgado I, Baer MR, Holleran JL, Egorin MJ (2009). Phase I study of vorinostat in combination with bortezomib for relapsed and refractory multiple myeloma. Clin Cancer Res.

[70] Siegel DS, Dimopoulos MA, Yoon SS, Laubach JP, Kaufman JL, Goldschmidt H, Reece DE, Leleu X, Durrant S, Offner FC, Cavo M, Nagler A, Jagannath S (2015). Vantage 095: vorinostat in combination with bortezomib in salvage multiple myeloma patients: final study results of a global phase 2b trial. Blood.

[71] Dimopoulos M, Siegel DS, Lonial S, Qi J, Hajek R, Facon T, Rosinol L, Williams C, Blacklock H, Goldschmidt H, Hungria V, Spencer A, Palumbo A (2013). Vorinostat or placebo in combination with bortezomib in patients with multiple myeloma (VANTAGE 088): a multicentre, randomised, double-blind study. Lancet Oncol.

[72] Richardson PG, Hungria VT, Yoon SS, Beksac M, Dimopoulos MA, Elghandour A, Jedrzejczak WW, Guenther A, Nakorn TN, Siritanaratkul N, Schlossman RL, Hou J, Moreau P (2016). Panobinostat plus bortezomib and dexamethasone in previously treated multiple myeloma: outcomes by prior treatment. Blood.

[73] San-Miguel JF, Hungria VT, Yoon SS, Beksac M, Dimopoulos MA, Elghandour A, Jedrzejczak WW, Günther A, Nakorn TN, Siritanaratkul N, Corradini P, Chuncharunee S, Lee JJ (2014). Panobinostat plus bortezomib and dexamethasone versus placebo plus bortezomib and dexamethasone in patients with relapsed or relapsed and refractory multiple myeloma: a multicentre, randomised, double-blind phase 3 trial. Lancet Oncol.

[74] Einsele H, Richardson P, Hungria V, Yoon SS, Beksac M, Dimopoulos M, Elghandour A, Jedrzejczak W, Guenther A, Nakorn TN, Siritanaratkul N, Schlossman R, Hou J (2015). Subgroup analysis by prior treatment among patients with relapsed or relapsed and refractory multiple myeloma in the panorama 1 study of panobinostat or placebo plus bortezomib and dexamethasone. EHA.

[75] van Rhee F, Szmania SM, Dillon M, van Abbema AM, Li X, Stone MK, Garg TK, Shi J, Moreno-Bost AM, Yun R, Balasa B, Ganguly B, Chao D (2009). Combinatorial efficacy of anti-CS1 monoclonal antibody elotuzumab (HuLuc63) and bortezomib against multiple myeloma. Mol Cancer Ther.

[76] Richardson PG, Moreau P, Jakubowiak AJ, Facon T, Jagannath S, Vij R, Reece DE, White DJ, Raab MS, Benboubker L, Rossi JF, Tsao C, Fry J (2015). Elotuzumab in combination with lenalidomide and dexamethasone in patients with relapsed multiple myeloma: interim results of a phase 2 study. Blood.

[77] Lonial S, Vij R, Harousseau JL, Facon T, Moreau P, Mazumder A, Kaufman JL, Leleu X, Tsao LC, Westland C, Singhal AK, Jagannath S (2012). Elotuzumab in combination with lenalidomide and low-dose dexamethasone in relapsed or refractory multiple myeloma. J Clin Oncol.

[78] Lonial S, Dimopoulos M, Palumbo A, White D, Grosicki S, Spicka I, Walter-Croneck A, Moreau P, Mateos MV, Magen H, Belch A, Reece D, Beksac M (2015). Elotuzumab therapy for relapsed or refractory multiple myeloma. N Engl J Med.

[79] Dimopoulos MA, Lonial S, White D, Moreau P, Palumbo A, San Miguel J, Shpilberg O, Anderson KC, Grosicki S, Spicka I, Walter-Croneck A, Magen-Nativ H, Mateos MV (2015). Eloquent-2 update: a phase 3, randomized, open-label study of elotuzumab in combination with lenalidomide/dexamethasone in patients with relapsed/refractory multiple myeloma - 3-year safety and efficacy follow-up. Blood.

[80] Jakubowiak AJ, Benson DM, Bensinger W, Siegel DS, Zimmerman TM, Mohrbacher A, Richardson PG, Afar DE, Singhal AK, Anderson KC (2012). Phase I trial of anti-CS1 monoclonal antibody elotuzumab in combination with bortezomib in the treatment of relapsed/refractory multiple myeloma. J Clin Oncol.

[81] Jakubowiak A, Offidani M, Pégourie B, De La Rubia J, Garderet L, Laribi K, Bosi A, Marasca R, Laubach J, Mohrbacher A, Carella AM, Singhal AK, Tsao LC (2016). Randomized phase 2 study of elotuzumab plus bortezomib/dexamethasone (Bd) versus Bd for relapsed/refractory multiple myeloma. Blood.

[82] Mateos MV, Granell M, Oriol A, Martinez-Lopez J, Blade J, Hernandez MT, Martín J, Gironella M, Lynch M, Bleickardt E, Paliwal P, Singhal A, San-Miguel J (2016). Elotuzumab in combination with thalidomide and low-dose dexamethasone: a phase 2 single-arm safety study in patients with relapsed/refractory multiple myeloma. Br J Haematol.

[83] van der Veer MS, de Weers M, van Kessel B, Bakker JM, Wittebol S, Parren PW, Lokhorst HM, Mutis T (2011). Towards effective immunotherapy of myeloma: enhanced elimination of myeloma cells by combination of lenalidomide with the human CD38 monoclonal antibody daratumumab. Haematologica.

[84] van der Veer MS, de Weers M, van Kessel B, Bakker JM, Wittebol S, Parren PW, Lokhorst HM, Mutis T (2011). The therapeutic human CD38 antibody daratumumab improves the anti-myeloma effect of newly emerging multi-drug therapies. Blood Cancer J.

[85] Plesner T, Arkenau HT, Lokhorst HM, Gimsing P, Krejcik J, Lemech C, Minnema MC, Lassen U, Laubach JP, Ahmadi T, Yeh H, Guckert ME, Feng H (2014). Safety and efficacy of daratumumab with lenalidomide and dexamethasone in relapsed or relapsed, refractory multiple myeloma. Blood.

[86] Dimopoulos MA, Oriol A, Nahi H, San-Miguel J, Bahlis NJ, Usmani SZ, Rabin N, Orlowski RZ, Komarnicki M, Suzuki K, Plesner T, Yoon SS, Ben Yehuda D (2016). Daratumumab, lenalidomide, and dexamethasone for multiple myeloma. N Engl J Med.

[87] Palumbo A, Chanan-Khan A, Weisel K, Nooka AK, Masszi T, Beksac M, Spicka I, Hungria V, Munder M, Mateos MV, Mark TM, Qi M, Schecter J (2016). Daratumumab, bortezomib, and dexamethasone for multiple myeloma. N Engl J Med.

[88] Avet-Loiseau H, Casneuf T, Chiu C, Laubach JP, Lee JJ, Moreau P, Plesner T, Nahi H, Khokhar NZ, Qi M, Schecter J, Carlton V, Qin X (2016). Evaluation of minimal residual disease (MRD) in relapsed/refractory multiple myeloma (RRMM) patients treated with daratumumab in combination with lenalidomide plus dexamethasone or bortezomib plus dexamethasone. Blood.

[89] Martin TG III, Baz R, Benson DM, Lendvai N, Campana F, Charpentier E, Vij R (2014). A phase Ib dose escalation trial of SAR650984 (Anti-CD-38 mAb) in combination with lenalidomide and dexamethasone in relapsed/refractory multiple myeloma. Blood.

[90] Görgün G, Samur MK, Cowens KB, Paula S, Bianchi G, Anderson JE, White RE, Singh A, Ohguchi H, Suzuki R, Kikuchi S, Harada T, Hideshima T (2015). Lenalidomide enhances immune checkpoint blockade-induced immune response in multiple myeloma. Clin Cancer Res.

[91] Mateos MV, Orlowski RZ, Siegel DS, Reece DE, Moreau P, Ocio EM, Shah JJ, Rodríguez-Otero P, Munshi NC, Avigan D, Ge JY, Marinello PM, San Miguel J (2016). Pembrolizumab in combination with lenalidomide and low-dose dexamethasone for relapsed/refractory multiple myeloma (RRMM): final efficacy and safety analysis. ASCO.

[92] Badros AZ, Kocoglu MH, Ma N, Rapoport AP, Lederer E, Philip S, Lesho P, Dell C, Hardy NM, Yared J, Goloubeva O, Singh Z (2015). A phase II study of anti PD-1 antibody pembrolizumab, pomalidomide and dexamethasone in patients with relapsed/refractory multiple myeloma (RRMM). Blood.

[93] Cavo M, Pantani L, Petrucci MT, Patriarca F, Zamagni E, Donnarumma D, Crippa C, Boccadoro M, Perrone G, Falcone A, Nozzoli C, Zambello R, Masini L (2012). Bortezomib-thalidomide-dexamethasone is superior to thalidomide-dexamethasone as consolidation therapy after autologous hematopoietic stem cell transplantation in patients with newly diagnosed multiple myeloma. Blood.

[94] Richardson PG, Weller E, Lonial S, Jakubowiak AJ, Jagannath S, Raje NS, Avigan DE, Xie W, Ghobrial IM, Schlossman RL, Mazumder A, Munshi NC, Vesole DH (2010). Lenalidomide, bortezomib, and dexamethasone combination therapy in patients with newly diagnosed multiple myeloma. Blood.

[95] Durie BG, Hoering A, Abidi MH, Rajkumar SV, Epstein J, Kahanic SP, Thakuri M, Reu F, Reynolds CM, Sexton R, Orlowski RZ, Barlogie B, Dispenzieri A (2017). Bortezomib with lenalidomide and dexamethasone versus lenalidomide and dexamethasone alone in patients with newly diagnosed myeloma without intent for immediate autologous stem-cell transplant (SWOG S0777): a randomised, open-label, phase 3 trial. Lancet.

[96] Moreau P, Hulin C, Caillot D, Marit G, Perrot A, Garderet L, Facon T, Benboubker L, Karlin L, Tiab M, Arnulf B, Fermand JP, Leleu X (2016). Ixazomib-lenalidomide-dexamethasone (IRd) combination before and after autologous stem cell transplantation (ASCT) followed by ixazomib maintenance in patients with newly diagnosed multiple myeloma (NDMM): a phase 2 study from the Intergroupe Francophone du MyéLome (IFM). Blood.

[97] Sonneveld P, Hacker E, Zweegman S, Kersten MJ, Vellenga E, van Marwijk-Kooy M, de Weerdt O, Lonergan S, Palumbo A, Lokhorst HM (2015). Carfilzomib combined with thalidomide and dexamethasone (CARTHADEX) as induction treatment prior to high-dose melphalan (HDM) in newly diagnosed patients with multiple myeloma (MM). A trial of the European Myeloma Network (EMN). Blood.

[98] Zimmerman T, Raje NS, Vij R, Reece D, Berdeja JG, Stephens LA, McDonnell K, Rosenbaum CA, Jasielec J, Richardson PG, Gurbuxani S, Nam J, Severson E (2016). Final results of a phase 2 trial of extended treatment (tx) with carfilzomib (CFZ), lenalidomide (LEN), and dexamethasone (KRd) plus autologous stem cell transplantation (ASCT) in newly diagnosed multiple myeloma (NDMM). Blood.

[99] Nooka AK, Lonial S (2016). New targets and new agents in high-risk multiple myeloma. Am Soc Clin Oncol Educ Book.

[100] Sonneveld P, Avet-Loiseau H, Lonial S, Usmani S, Siegel D, Anderson KC, Chng WJ, Moreau P, Attal M, Kyle RA, Caers J, Hillengass J, San Miguel J (2016). Treatment of multiple myeloma with high-risk cytogenetics: a consensus of the International Myeloma Working Group. Blood.

[101] Calimeri T, Ferreri AJ (2017). m-TOR inhibitors and their potential role in haematological malignancies. Br J Haematol.

[102] Günther A, Baumann P, Burger R, Kellner C, Klapper W, Schmidmaier R, Gramatzki M (2015). Activity of everolimus (RAD001) in relapsed and/or refractory multiple myeloma: a phase I study. Haematologica.

[103] Yee AJ, Hari P, Marcheselli R, Mahindra AK, Cirstea DD, Scullen TA, Burke JN, Rodig SJ, Hideshima T, Laubach JP, Ghobrial IM, Schlossman RL, Munshi NC (2014). Outcomes in patients with relapsed or refractory multiple myeloma in a phase I study of everolimus in combination with lenalidomide. Br J Haematol.

[104] Hofmeister CC, Yang X, Pichiorri F, Chen P, Rozewski DM, Johnson AJ, Lee S, Liu Z, Garr CL, Hade EM, Ji J, Schaaf LJ, Benson DM (2011). Phase I trial of lenalidomide and CCI-779 in patients with relapsed multiple myeloma: evidence for lenalidomide-CCI-779 interaction via P-glycoprotein. J Clin Oncol.

[105] Ghobrial IM, Weller E, Vij R, Munshi NC, Banwait R, Bagshaw M, Schlossman R, Leduc R, Chuma S, Kunsman J, Laubach J, Jakubowiak AJ, Maiso P (2011). Weekly bortezomib in combination with temsirolimus in relapsed or relapsed and refractory multiple myeloma: a multicentre, phase 1/2, open-label, dose-escalation study. Lancet Oncol.

[106] Gilmartin AG, Bleam MR, Groy A, Moss KG, Minthorn EA, Kulkarni SG, Rominger CM, Erskine S, Fisher KE, Yang J, Zappacosta F, Annan R, Sutton D (2011). GSK1120212 (JTP-74057) is an inhibitor of MEK activity and activation with favorable pharmacokinetic properties for sustained in vivo pathway inhibition. Clin Cancer Res.

[107] Tolcher AW, Patnaik A, Papadopoulos KP, Rasco DW, Becerra CR, Allred AJ, Orford K, Aktan G, Ferron-Brady G, Ibrahim N, Gauvin J, Motwani M, Cornfeld M (2015). Phase I study of the MEK inhibitor trametinib in combination with the AKT inhibitor afuresertib in patients with solid tumors and multiple myeloma. Cancer Chemother Pharmacol.

[108] O'Donnell E, Raje NS (2013). Targeting BRAF in multiple myeloma. Cancer Discov.

[109] Mey UJ, Renner C, von Moos R (2016). Vemurafenib in combination with cobimetinib in relapsed and refractory extramedullary multiple myeloma harboring the BRAF V600E mutation. Hematol Oncol.

[110] Raje N, Chau I, Hyman DM, Ribrag V, Blay JY, Tabernero J, Elez-Fernandez ME, Wolf JS, Sirzen F, Yee A, Faris J, Kaiser M, Landau H (2015). Vemurafenib (VEM) in relapsed refractory multiple myeloma harboring BRAF V600 mutations (V600m): a cohort of the histology-independent VE-basket study. Blood.

[111] Kennedy SG, Kandel ES, Cross TK, Hay N (1999). Akt/Protein kinase B inhibits cell death by preventing the release of cytochrome c from mitochondria. Mol Cell Biol.

[112] Brunet A, Bonni A, Zigmond MJ, Lin MZ, Juo P, Hu LS, Anderson MJ, Arden KC, Blenis J, Greenberg ME (1999). Akt promotes cell survival by phosphorylating and inhibiting a Forkhead transcription factor. Cell.

[113] Wendel HG, De Stanchina E, Fridman JS, Malina A, Ray S, Kogan S, Cordon-Cardo C, Pelletier J, Lowe SW (2004). Survival signalling by Akt and eIF4E in oncogenesis and cancer therapy. Nature.

[114] Spencer A, Yoon SS, Harrison SJ, Morris SR, Smith DA, Brigandi RA, Gauvin J, Kumar R, Opalinska JB, Chen C (2014). The novel AKT inhibitor afuresertib shows favorable safety, pharmacokinetics, and clinical activity in multiple myeloma. Blood.

[115] Bataille R, Barlogie B, Lu ZY, Rossi JF, Lavabre-Bertrand T, Beck T, Wijdenes J, Brochier J, Klein B (1995). Biologic effects of anti-interleukin-6 murine monoclonal antibody in advanced multiple myeloma. Blood.

[116] Voorhees PM, Manges RF, Sonneveld P, Jagannath S, Somlo G, Krishnan A, Lentzsch S, Frank RC, Zweegman S, Wijermans PW, Rijnbeek B, Qin X, Cornfeld MJ (2015). A phase 2 multicenter study of siltuximab, an anti-IL-6 monoclonal antibody, in patients with relapsed or refractory multiple myeloma. Blood.

[117] Orlowski RZ, Gercheva L, Williams C, Sutherland H, Robak T, Masszi T, Goranova-Marinova V, Dimopoulos MA, Cavenagh JD, Špička I, Maiolino A, Suvorov A, Bladé J (2011). A phase 2, randomized, double-blind, placebo-controlled study of siltuximab (anti-IL-6 mAb) and bortezomib versus bortezomib alone in patients with relapsed or refractory multiple myeloma. Am J Hematol.

[118] Suzuki K, Ogura M, Abe Y, Suzuki T, Tobinai K, Ando K, Taniwaki M, Maruyama D, Kojima M, Kuroda J, Achira M, Iizuka K (2015). Phase 1 study in Japan of siltuximab, an anti-IL-6 monoclonal antibody, in relapsed/refractory multiple myeloma. Int J Hematol.

